# Fabrication of a Disposable Electrochemical Immunosensor Based on Nanochannel Array Modified Electrodes and Gated Electrochemical Signals for Sensitive Determination of C-Reactive Protein

**DOI:** 10.3390/nano12223981

**Published:** 2022-11-11

**Authors:** Ning Ma, Xuan Luo, Weidong Wu, Jiyang Liu

**Affiliations:** 1 Shanxi Bethune Hospital, Shanxi Academy of Medical Sciences, Tongji Shanxi Hospital, Third Hospital of Shanxi Medical University, Taiyuan 030032, China; 2 Tongji Hospital, Tongji Medical College, Huazhong University of Science and Technology, Wuhan 430030, China; 3 Department of Chemistry, Key Laboratory of Surface & Interface Science of Polymer Materials of Zhejiang Province, Zhejiang Sci-Tech University, Hangzhou 310018, China

**Keywords:** electrochemical immunosensor, disposable and integrated electrode, vertically-ordered mesoporous silica-nanochannel film, screen-printed carbon electrode, c-reactive protein

## Abstract

Sensitive determination of C-reactive protein (CRP) is of great significance because it is an early indicator of inflammation in cardiovascular disease and acute myocardial infarction. A disposable electrode with an integrated three-electrode system (working, reference, and counter electrodes) has great potential in the detection of biomarkers. In this work, an electrochemical immunosensing platform was fabricated on disposable and integrated screen-printed carbon electrode (SPCE) by introducing nanochannel arrays and gated electrochemical signals, which can achieve the sensitive detection of CRP in serum. To introduce active reactive groups for the fabrication of immuno-recognitive interface, vertically-ordered mesoporous silica-nanochannel film (VMSF) with rich amino groups (NH_2_-VMSF) was rapidly grown by electrochemical assisted self-assembly (EASA). The electrochemically reduced graphene oxide (ErGO) synthesized in situ during the growth of NH_2_-VMSF was used as a conductive adhesive glue to achieve stable bonding of the nanochannel array (NH_2_-VMSF/ErGO/SPCE). After the amino group on the outer surface of NH_2_-VMSF reacted with bifunctional glutaraldehyde (GA/NH_2_-VMSF/ErGO/SPCE), the converted aldehyde surface was applied for covalent immobilization of the recognitive antibody (Ab) followed with the blocking of the non-specific sites. The fabricated immunosensor, Ab/GA/NH_2_-VMSF/ErGO/SPCE, enables sensitive detection of CRP in the range from 10 pg/mL to 100 ng/mL with low limit of detection (LOD, 8 pg/mL, S/N = 3). The immunosensor possessed high selectivity and can realize reliable determination of CRP in human serum.

## 1. Introduction

C-reactive protein (CRP) is an acute protein that rises sharply in the plasma when the body is infected or tissue is damaged [1,2]. CRP plays an important protective role in the body’s natural immune process because it can activate complement and enhance the phagocytic function of phagocytes to remove pathogenic microorganisms that invade the body, as well as damaged, necrotic, and apoptotic tissue cells. More importantly, CRP is designated as an early indicator of inflammation in cardiovascular diseases and acute myocardial infarction [3,4]. For instance, CRP is directly involved in atherosclerosis. It has been demonstrated that large amounts of CRP are deposited in human atherosclerotic plaques. Therefore, the highly sensitive and specific determination of CRP is of great significance for clinical diagnosis [5,6]. Up to now, the methods for the detection of CRP include immunodiffusion, radioimmunosensor, turbidimetric method, enzyme or fluorescence labeled immunosensor, electrochemistry (EC) or electrochemiluminescence (ECL), etc. [3,7,8,9,10,11,12,13]. However, these strategies often have the problems of complex detection processes or insufficient sensitivity. An electrochemical immunosensor is an analytical method that combines immunosensors with electrochemical detection technology. As known, compared with other detection methods (such as optical technology, etc.), electrochemical detection has the advantages of simple operation, rapid detection, high sensitivity, and simple instrumentation [14,15,16,17]. Owing to the high specificity and selectivity of immune recognition and the unique merits of electrochemical detection, electrochemical immunosensors have great application potential in the clinical detection of CRP.

Effective construction of an immune recognition interface with biological recognition is the basis of an electrochemical immunosensor, which is usually fabricated on the surface of the supporting electrode. The most common electrochemical electrodes include noble metal electrodes (e.g., gold or platinum electrodes, etc.), carbon electrodes (e.g., glassy carbon electrode-GCE), etc. [18,19]. Yet these electrodes are very expensive and require a tedious grinding process before use. Compared to these electrodes, disposable electrodes offer significant advantages including high repeatability between each electrode, no need for electrode maintenance, flexibility, and customizable electrode structure [20,21,22,23]. Screen-printed carbon electrodes (SPCEs) are the most commonly used disposable electrodes and have the advantages of simple structure, low cost, easy large-scale production, and flexible design, demonstrating potential applications in electrochemical sensing, photovoltaic power, environmental detection, clinical analysis, food detection, and other fields [24,25,26,27]. Generally, three electrodes are printed on the SPCE substrate, namely the working electrode (WE), the reference electrode (RE), and the auxiliary electrode (AE), leading to a classic electrochemical three-electrode system. Fabrication of the electrochemical immunosensor on SPCE and its application in the detection of CRP is highly desirable.

The accuracy of electrochemical detection is also the key to electrochemical immunosensor. The biggest challenge is the contamination of the electrode surface, which is more prominent in the analysis of complex biological samples (e.g., serum). For example, a large number of proteins contained in serum easily adhered to the electrodes through non-specific adsorption, which reduces the electrochemically active area of the electrode and results in low detection accuracy and stability. Therefore, it is very important to improve the anti-fouling ability of the electrode. Until now, the anti-fouling strategies for electrodes can be commonly divided into two categories. One is by introducing charged hydrophilic polymers [28,29]. The principle of this strategy lies in the reduced non-specific adsorption of proteins by increasing the hydrophilicity of the electrode surface and the exclusion of species with the same charges through electrostatic repulsion. Another strategy is to introduce functional nanomaterials on the electrode surface [30,31]. This strategy is more advantageous due to the rich structure, easy modification, and abundant function of nanomaterials. Amongst, the introduction of vertically-ordered mesoporous silica nanomembrane film (VMSF) on the surface of electrodes has attracted much attention in recent years [32,33,34]. VMSF has ultra-small size (2–3 nm in pore size), high density, and parallel nanochannel array, which ensure the permeability of small molecules to the underlying electrode. In addition, ultra-small nanochannels can effectively exclude substances such as proteins through size sieving [35,36]. Combined with the non-conductivity property of its silica structure, VMSF can effectively prevent the contamination of the underlying conductive electrodes [37,38,39]. In addition, although the nanochannel cannot accommodate bio-recognitive ligands (e.g., protein, DNA, etc.), the outer surface of VMSF, that is, the entrance of nanochannels can be modified by bioligand molecules, leading to significant gated effects [40,41]. For example, the formation of antigen-antibody complexes can significantly alter the diffusion of solution-based electrochemical redox probes in VMSF. Combined with the advantages of easy preparation and low cost of VMSF, VMSF-modified electrodes are promising in signal-gating electrochemical immunosensors.

In this work, a disposable and integrated electrochemical immunosensor was fabricated for sensitive determination of C-reactive protein based on a nanochannel array modified electrode and gated electrochemical signal. Screen-printed electrode (SPCE) was used as the disposable supporting electrode with an integrated three-electrode system. To improve the binding stability of VMSF on SPCE, electrochemically reduced graphene oxide (ErGO) formed in situ during VMSF growth was used as the conductive bonding layer. Using siloxanes containing amino groups as precursors, VMSF containing amino groups (NH_2_-VMSF) was rapidly grown by the simple electrochemical-assisted self-assembly (EASA) method. When the outer surface of VMSF was derivatized with glutaraldehyde (GA), the aldehyde-based surface enabled covalent immobilization of CRP antibody (Ab). The immuno-electrode obtained after blocking the non-specific sites using bovine serum albumin (BSA) can realize sensitive detection of CRP because the formation of immuno-complexes blocked the nanochannels and reduced the diffusion of solution-phase redox probes (Fe(CN)_6_^3−/4−^), resulting in a gated electrochemical signal.

## 2. Materials and Methods

### 2.1. Chemicals and Materials

Graphene oxide (GO) was synthesized by the conventional Hummers method [42,43]. Tetraethyl orthosilicate (TEOS), 3-aminopropyltriethoxysilane (APTES), hexadecyl trime-thyl ammonium bromide (CTAB), potassium ferricyanide (K_3_[Fe(CN)_6_]), sodium phosphate dibasic dodecahy-drate (Na_2_HPO_4_•12H_2_O), bovine whole blood albumin (BSA), glutaraldehyde(GA), and potassium hydrogen phthalate (KHP) were all purchased from Aladdin Chemistry Co., Ltd. (Shanghai, China). Potassium chloride (KCl), sulfuric acid (H_2_SO_4_), and ethanol (EtOH) were obtained from Hangzhou Gaojing Chemistry Co., Ltd. (Hangzhou, China). Sodium dihydrogen phosphate dehydrate (NaH_2_PO_4_•2H_2_O) and 3-aminopropyltriethoxysilane (APTES) were purchased from Macklin (Shanghai, China). Hydrochloric acid (HCl) was obtained from Hangzhou Shuanglin Chemical Reagent Co., Ltd. C-reactive protein (CRP), CRP antibody, serum amyloid A (SAA), alpha-fetoprotein (AFP), and procalcitonin (PCT) were bought from Nanjing OkayBio (Nanjing, China). Carcinoembryonic antigen (CEA) was purchased from Beijing KEY-BIO Biotech Co., Ltd. (Beijing, China). A screen-printed carbon electrode (SPCE) with a three-electrode system (DRP-C110-U75) was obtained from Metrohm (Heilissau, Switzerland). Briefly, the carbon electrode was used as the working electrode and the counter electrode (4 mm diameter), and the silver electrode was used as the reference electrode. All chemicals used were of analytical grade and used without further treatment. The ultrapure water (18.2 MΩ•cm) used in the experiment was prepared by Mill-Q Systems (Millipore Company, Billerica, MA, USA).

### 2.2. Measurements and Instrumentations

X-ray photoelectron spectroscopy (XPS) analysis of GO or ErGO-modified SPCEs was performed using Mg Kα irradiation at 250 W and 14 kV (PHI5300, PE Ltd., Boston, MA, USA). The morphology of NH_2_-VMSF was investigated by transmission electron microscopy (TEM) using an HT7700 microscope (Hitachi, Tokyo, Japan) with copper mesh as the support material. An accelerating voltage of 100 kV was applied. Before measurement, NH_2_-VMSF was carefully scraped from the electrode surface and sonicated in ethanol, to obtain the sample dispersion. All electrochemical experiments including cyclic voltammetry (CV), electrochemical impedance spectroscopy (EIS), and differential pulse voltammetry (DPV) were performed on an Autolab electrochemical workstation (PGSTAT302N, Metrohm, Heilissau, Switzerland). For EIS measurement, frequencies ranged from 0.1 Hz to 100 kHz with a perturbation amplitude of 5 mV.

### 2.3. Preparation of NH_2_-VMSF Modified SPCE

Before use, SPCE was electrochemically polished 10 times by cyclic voltammetry scan in diluted sulfuric acid solution (0.05 M). The potential was from 0.4 V to 1.0 V. Then, the electrode was thoroughly rinsed with ultrapure water and dried with a stream of N_2_. To modify the electrode, GO dispersion (0.1 mg/mL, 10 μL) was dropped on the WE area of SPCE and dried naturally at room temperature. Then, NH_2_-VMSF was synthesized in the presence of CTAB micelles (SM) using mixed siloxane precursors containing APTES and TEOS [44]. Specifically, CTAB (1.585 g) and APTES (0.159 g) were added to a mixture containing ethanol (20 mL) and an equal volume of NaNO_3_ solution (0.1 M, pH = 2.6) under stirring. After the pH of the solution was adjusted to 3.0 with HCl (6 M), TEOS (2.891 g) was added and the obtained mixture was stirred at room temperature for 2.5 h. When the SPCE was placed in the above precursor solution, a current of −350 μA was applied to the working electrode for 10 s. After thorough rinsing with ultrapure water, the electrode containing surfactant SM (SM@NH_2_-VMSF/ErGO/SPCE) was obtained. After the electrodes were aged at 80 °C for 10 h, SM was removed using 0.1 M HCl/EtOH solution (*V*:*V* = 1:1) to obtain electrodes with open nanochannels (NH_2_-VMSF/ErGO/SPCE). To prove the role of nanochannels, a control electrode containing an amino group but without nanochannels was prepared and denoted as NH_2_-ErGO/SPCE. Briefly, ErGO/SPCE was prepared in the solution without siloxane precursor under the same conditions. The dried ErGO/SPCE was immersed in an acetone (48 mL) solution containing APTES (1.136 g) for 1 h. Then, NH_2_-ErGO/SPCE was obtained after successively washing with acetone, ethanol, acetone, and water, respectively.

### 2.4. Fabrication of the Immuonsensor

To obtain the aldehyde-modified surface, the NH_2_-VMSF/ErGO/SPCE electrode was immersed in 2% GA (pH = 7.4) solution for 1 h at 37 °C in the dark. After thoroughly washing with phosphate buffer solution (PBS, 0.05 M, pH = 7.0), the electrode was soaked in CRP antibody (50 μg/mL) solution and incubated at 37 °C for 1 h. After the unbound antibody was rinsed with PBS, the obtained electrode was immersed in BSA solution (1 mg/mL) to block the non-specific sites. The resulting immunoelectrode was designated as Ab/NH_2_-VMSF/ErGO/SPCE. For the incubation, the electrode was inserted into the antigen solution, and then the reaction system was placed in an incubator with constant temperature (37 °C) and humidity (90%) for 1 h.

### 2.5. Electrochemical Determination of CRP

The Ab/NH_2_-VMSF/ErGO/SPCE immunosensors were incubated with different concentrations of CRP for 60 min at 37 °C. Using PBS (0.05 M, pH = 7.0) containing Fe(CN)_6_^3−/4−^ (1.25 mM) and KCl (0.1 M) as the detection solution, the electrochemical signals on the immunoelectrodes before and after CRP binding (37 °C, 1 h) were measured. For actual sample analysis, CRP in human serum was determined using a standard addition method. Serum samples were diluted by a factor of 50 before detection. To evaluate the stability in storage, the immunosensor was stored in PBS (0.05 M, pH = 7.0) at 4 °C.

## 3. Results and Discussion

### 3.1. Fabrication of Electrochemical Immunosensor on NH_2_-VMSF Modified SPCE

**Figure 1:**
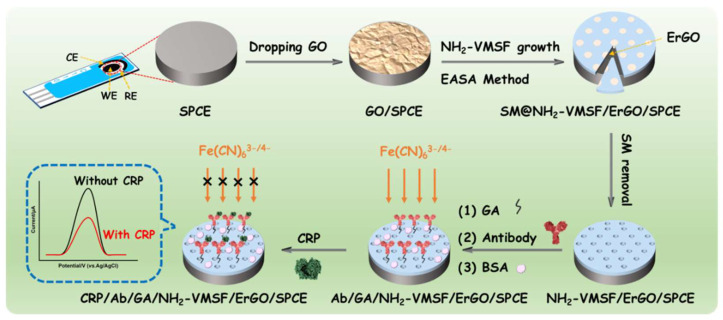
Schematic illustration for fabrication of electrochemical immunosensor on NH_2_-VMSF modified SPCE and the detection of CRP based on gated electrochemical signal.

VMSF has a uniformly sized, high-density nanochannel array. In addition, functional chemical reactive groups can be easily introduced using functional siloxane precursors. However, it is difficult to stably bind VMSF directly to carbon electrodes. Graphene materials exhibit different dimensionals (e.g., 0D grpahene quantum dots [45,46,47,48], 2D graphene sheets [49,50], and 3D graphene foams [51,52,53]) and have been employed as functional elements to improve the sensitivity of (bio)sensors. Herein, electrochemically reduced graphene oxide (ErGO) formed in situ during VMSF growth was used as the conductive bonding layer to improve the bonding stability of VMSF on SPCE. To facilitate the covalent immobilization of the recognitive antibody (Ab), VMSF-containing amino groups (NH_2_-VMSF) were prepared by the EASA method with amino group-containing siloxane being the precursor. As illustrated in Figure 1, graphene oxide (GO) was first dropped on the surface of SPCE. The mechanism of the EASA method lies in the kinetic control of the sol-gel process of siloxane precursors around surfactant micelle (SM) templates. To grow NH_2_-VMSF, a negative current was applied on the electrode to increase the pH in situ on the electrode surface through reducing protons, which promoted the hydrolysis and condensation of siloxane precursors around the SM template. During this process, GO drop-coated on the surface of SPCE can be electrochemically reduced in situ, leading to electrochemically reduced graphene oxide (ErGO). On the one hand, ErGO interacted with the underlying carbon electrode through hydrophobic interaction. On the other hand, the oxygen-containing groups on ErGO can undergo a condensation reaction with the silanol groups in NH_2_-VMSF, realizing covalent bonding. Thus, ErGO acted as the conductive binding layer to improve the binding stability of NH_2_-VMSF. 

When VMSF was grown, the resulting nanochannels were blocked by SM and the electrode was denoted as SM@NH_2_-VMSF/ErGO/SPCE. After SM was removed, the obtained NH_2_-VMSF/ErGO/SPCE electrode had an open nanochannel. Since the outer surface of NH_2_-VMSF contained chemically reactive amino groups, the aldehyde-based surface (GA/NH_2_-VMSF/ErGO/SPCE) was easily achieved by reacting NH_2_-VMSF with the bifunctional group reagent glutaraldehyde (GA), which can realize covalent immobilization of the recognitive antibody (Ab) through the reaction between aldehyde group and amino group in Ab. After blocking non-specific sites with BSA, the immunosensor (Ab/GA/NH_2_-VMSF/ErGO/SPCE) was finally obtained. In comparison with the orientated immobilization strategy that commonly applied biotin and streptavidin, this immobilization of the antibody is simple, fast, and low-cost. Due to the high density of nanochannels in NH_2_-VMSF, redox probes (Fe(CN)_6_^3−/4−^) in a solution can efficiently diffuse to the underlying electrode, generating electrochemical signals. When the fabricated immunosensor is incubated with the antigen, the formed antibody-antigen complex with a large size will block the entrance of some nanochannel because of its large size, leading to the decreased mass transport of Fe(CN)_6_^3−/4−^ in the solution to the underlying electrode. As a result, the detected electrochemical signal decreased. This phenomenon is the gated effect. Based on this principle, the constructed immunosensor can realize sensitive electrochemical detection of CRP.

### 3.2. Characterization of NH_2_-VMSF and ErGO

**Figure 2:**
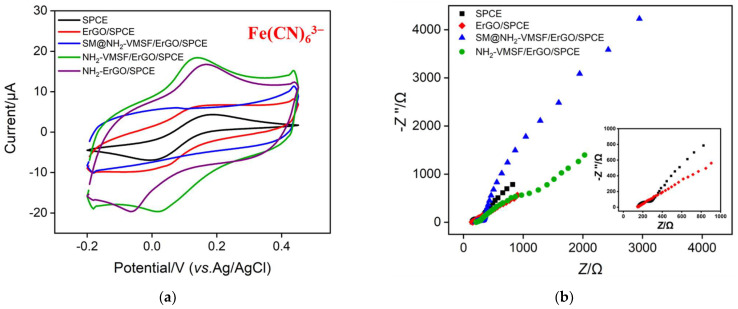
(**a**) Cyclic voltammetry (CV) curves obtained on different electrodes in 0.05 M KHP containing 0.5 mM Fe(CN)_6_^3−^. (**b**) EIS curves obtained on different electrodes. The electrolyte solution is 2.5 mM Fe(CN)_6_^3−/4−^ containing 0.1 M KCl, The inset is the magnified EIS plots on SPCE and ErGO/SPCE.

The changes in the electrode surface during the growth of NH_2_-VMSF were investigated using a standard electrochemical probe (Fe(CN)_6_^3−^). As shown in Figure 2a, a reversible electrochemical redox peak of Fe(CN)_6_^3−^ was observed on the supporting SPCE. When NH_2_-VMSF growth was completed, no obvious Faraday current was measured on the resulting SM@NH_2_-VMSF/ErGO/SPCE. This is attributed to the inhibition of probe diffusion to the underlying electrode due to the blocking of the nanochannels by SM. This phenomenon also demonstrated that NH_2_-VMSF had no cracks or fractured regions, otherwise the redox probes would directly interact with the electrode to generate electrochemical signals. When SM was removed, the opening of the nanochannels resulted in a significant electrochemical signal of Fe(CN)_6_^3−^ on NH_2_-VMSF/ErGO/SPCE. In addition, the oxidation and reduction peak currents are higher than those obtained on SPCE. This is attributed to the presence of NH_2_ groups in the nanochannels, which act as cationic sites to enrich negatively charged Fe(CN)_6_^3−^, resulting in increased peak currents. When NH_2_-VMSF was removed by sodium hydroxide, the redox peak of the probe could also be detected on ErGO/SPCE. The larger charging current than that of SPCE, proving the increase of electrode active area. Figure 2a also shows CV curves of Fe(CN)_6_^3−^ on ErGO/SPCE, NH_2_-ErGO/SPCE, and NH_2_-VMSF/ErGO/SPCE. As seen, NH_2_-ErGO/SCPE has a higher redox peak current compared with ErGO/SPCE, which is attributed to the fact that the amino group can enrich the negatively charged Fe(CN)_6_^3−^ through electrostatic interaction. However, NH_2_-VMSF/ErGO/SPCE with grown nanochannels exhibits the highest peak current, proving that the nanochannels rich in amino groups can significantly enrich Fe(CN)_6_^3−^. In addition, the peak-to-peak difference decreases, indicating the improved reversibility of the electrochemical process of Fe(CN)_6_^3−^.

The changes in the electrode interface during this process were also demonstrated by electrochemical impedance spectroscopy (EIS) characterization. Figure 2b shows the EIS curves obtained on the above electrodes. Each EIS curve consists of a semicircle in the high- frequency region and a linear part in the low-frequency region, where the former represents an electron transfer-limited process and the latter is a diffusion-limited process. The equivalent diameter of the semicircle in the high-frequency region corresponds to the charge transfer resistance (*R*_ct_). As seen, the modification of ErGO on SPCE significantly reduced the *R*_ct_ of the electrode. When the nanochannels of NH_2_-VMSF were blocked by SM, no diffusion-limited process of the electrode was observed on the EIS curve in the tested frequency range. In comparison, the following opening of nanochannels after SM removal resulted in a significant reduction in *R*_ct_. The function of nanochannels was investigated using the control electrode containing an amino group but without nanochannels, which was prepared by amino functionalization of ErGO/SPCE and denoted as NH_2_-ErGO/SPCE.

**Figure 3:**
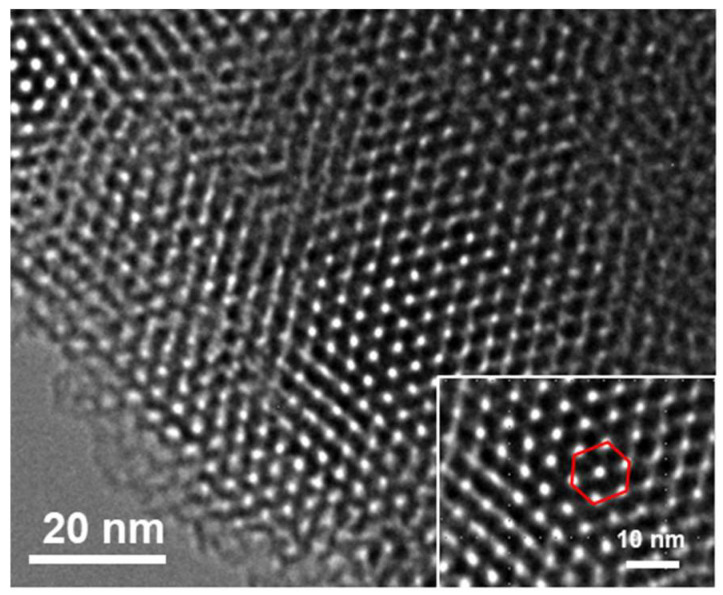
Top-view TEM image of NH_2_-VMSF at different magnification.

Transmission electron microscopy (TEM) was applied to investigate the morphology of NH_2_-VMSF. As shown in Figure 3, NH_2_-VMSF has an ordered silica nanoporous structure. No rupture was observed on NH_2_-VMSF over larger areas. The high-resolution TEM image (HRTEM, inset in Figure 3) revealed a hexagonal stacked structure with a pore size of about 2–3 nm.

**Figure 4:**
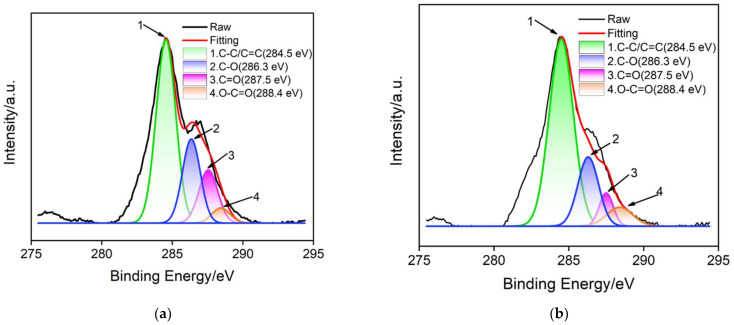
High-resolution C 1 s XPS spectra of GO/SPCE (**a**) or ErGO/SPCE (**b**) electrode.

An X-ray photoelectron spectroscopy (XPS) was used to further verify that GO was electrochemically reduced to ErGO in situ during the growth of NH_2_-VMSF. Figure 4a and 4b show the high-resolution C1s spectra of GO/SPCE and ErGO/SPCE, respectively. As shown, C-C/C=C peak of ErGO/SPCE significantly enhanced in comparison with that of GO/SPCE, indicating the increase of sp^2^ carbon structure in graphene. This phenomenon proves that GO on the surface of SPCE is reduced to ErGO during the growth of NH_2_-VMSF. In addition, the appearance of C-O, C=O, O-C=O groups revealed the existence of oxygen-containing groups on ErGO. These oxygen-containing groups will be beneficial to improve the stability of NH_2_-VMSF because they can form covalent bonds through condensation reactions with silanol groups in NH_2_-VMSF. Therefore, ErGO can act as a conductive adhesive layer and realize stable modification of NH_2_-VMSF on SPCE. On the one hand, ErGO can stably bind to SPCE through hydrophobic or π-π interaction. At the same time, NH_2_-VMSF can be stably bound to ErGO.

### 3.3. Fabrition of the Immunosensor

**Figure 5:**
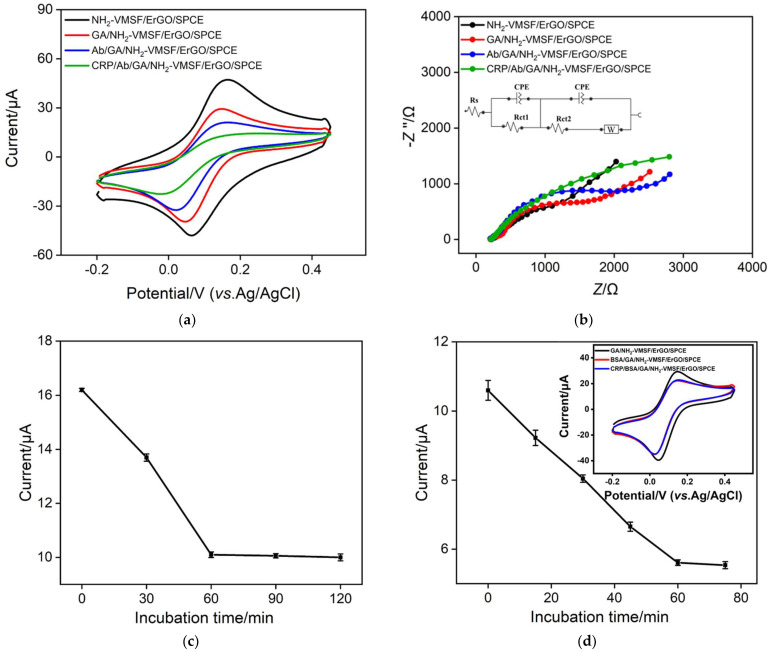
(**a**) CV curves obtained on different electrodes obtained in the fabrication of the immunosensor in 0.1 M KCl containing 1.25 mM Fe(CN)_6_^3−^. (**b**) EIS curves obtained on different electrodes obtained in the fabrication of the immunosensor. The electrolyte solution is 2.5 mM Fe(CN)_6_^3−/4−^ containing 0.1 M KCl. Inset is the illustration for the equivalent circuit. (**c**) Effect of incubation time between Ab on the aldehyde surface on the current response of the proposed immunosensor. (**d**) Effect of incubation time between the immuno-recognitive surface and the detected CRP on the current response of the proposed immunosensor. The error bars represent the relative standard deviation (RSD) of three measurements. Inset in (**d**) is CV curve obtained on different electrodes. The electrolyte solution is 2.5 mM Fe(CN)_6_^3−/4−^ containing 0.1 M KCl.

Effective immobilization of antibodies is the key to constructing an immunosensing system. Although the nanochannel of NH_2_-VMSF cannot accommodate the entry of antibodies owing to ultra-small pore size, the outer surface of NH_2_-VMSF (also the entrance of the nanochannel) can serve as the space for antibody immobilization. As illustrated in Figure 1, the aldehyde group derivatization of the amino group in NH_2_-VMSF produces an aldehyde group surface that can achieve covalent immobilization of antibodies. Electrochemical methods including cyclic voltammetry (CV) and EIS were also employed to study the changes in the electrode surface during the construction of immunosensor. Figure 5a shows the CV curves of different electrodes in the [Fe(CN)_6_]^4−/3−^ probe. As demonstrated, the reaction of GA with NH_2_-VMSF leads to a decrease in the electrochemical signal. This might be ascribed that the reaction between amino group and aldehyde group reduces the number of an amino group, leading to decreased electrostatic interaction towards negatively charged Fe(CN)_6_^3−/4−^ and reduced electrochemical signal. When Ab was covalently immobilized on the aldehyde-modified surface and the non-specific binding sites of the electrode were blocked by BSA, the electrochemical signal of the resulting immunoelectrode (Ab/NH_2_-VMSF/ErGO/SPCE) significantly reduced. This is attributed to the fact that both the antibody and BSA are proteins, and their non-conductivity leads to an increase in the interfacial resistance of the electrodes. When the immunoelectrode was incubated with CRP, the electrochemical signal of the probe further decreased, proving that the formed antigen-antibody complex further blocked the nanochannel. The EIS measurements shown in Figure 5b also demonstrate the same experimental conclusion. Compared with the typical Randles equivalent circuit model that contains one constant-phase element (CPE), the modified Randles equivalent circuit model with two CPEs is more accurate and has been extensively used to approximate the EIS data of film electrodes [54]. Therefore the Nyquist plots in Figure 5b were fitted to the modified Randles equivalent circuit model [13,55]. As illustrated in inset of Figure 5b, two CPEs, two charge-transfer resistances (*R*ct), Warburg impedance (W), and the resistance of the electrolyte solution (Rs) are included, where *R*ct_1_ and *R*ct_2_ correspond to the film resistance and charge-transfer kinetics at electrodes, respectively. Compared with *R*ct_1_, *R*ct_2_ has great value for quantitative information because of its significant increases with the modification. Derivatization of GA, immobilization of Ab, and blocking of non-specific sites all resulted in a continuous increase in *R*ct_2_ (9.59 Ω for NH_2_-VMSF/ErGO/SPC, 1.08 KΩ for GA/NH_2_-VMSF/ErGO/SPCE, 1.41 KΩ for Ab/GA/NH_2_-VMSF/ErGO/SPCE). When the immunoelectrode was incubated with CRP, the *R*ct_2_ further increased (3.52 KΩ), indicating the successful capture of CRP on the immuno-recognitive interface.

To further improve the performance of the immunosensor, the time for the covalent immobilization of antibodies during the preparation of the immunoelectrode was optimized. When the antibody reacted with the aldehyde-modified surface at different times, the resulting immunoelectrode was incubated with CRP. The peak currents of each electrode after being bound to CRP were shown in Figure 5c. As shown, the increase in the reaction time resulted in a decrease of the peak current, indicating that the amount of the immobilized antibody and the following bound CRP increased. When the immobilization time was 60 min, the electrochemical signal became stable, proving that the immobilization of antibodies on an aldehyde-based surface reached saturation. In addition, the effect of the binding time of CRP on the immuno-recognitive interface was also investigated. It can be found that the electrochemical signal reached a plateau when the antigen-antibody was 60 min, proving the saturated binding of the antigen (Figure 5d). To investigate the non-specific binding of antigen to the surface, BSA was immobilized on GA/NH_2_-VMSF/ErGO/SPCE and the obtained electrode was denoted as BSA/GA/NH_2_-VMSF/ErGO/SPCE. As shown in the inset of Figure 5d, BSA/GA/NH_2_-VMSF/ErGO/SPCE exhibited almost no change in CV signal in the absence or presence of CRP, indicating no non-specific binding of antigen to the surface.

### 3.4. Sensitive Determination of CRP Using the Developed Immunosensor

**Figure 6:**
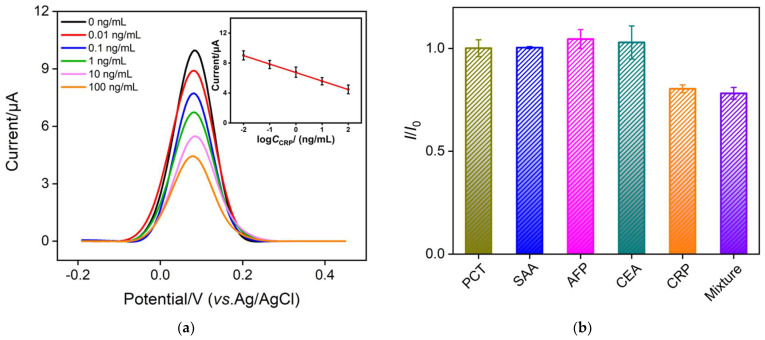
(**a**) Differential pulse voltametric curves of the immunosensor to various concentrations of CRP. Inset was the corresponding calibration curve for the detection of CRP. (**b**) The ratio of peak current when the immunosensor was incubated with different protein or their mixture. *I*_0_ and *I* were the peak current before and after the incubation. The concentration of each protein was 1 ng/mL. The mixture contained CRP (1 ng/mL) and all other proteins (each concentration was 1 ng/mL). The immunosensors were incubated with different protein or their mixture for 60 min at 37 °C. The error bars represent the relative standard deviation (RSD) of three measurements.

When the antibody on the immune-recognitive specifically binds to CRP, the formed antigen-antibody immunocomplex will hinder the diffusion of the redox probe to the underlying electrode. Based on this gated electrochemical signal, the constructed immunosensor can be used for the electrochemical detection of CRP. Figure 6a shows the differential pulse voltammetry (DPV) curves obtained when the immunosensors incubate with different concentrations of CRP. As seen, the increase in CRP concentration leads to the decreased electrochemical signal of the redox probe. The peak current (*I,* μA) was linear proportional to the logarithm of the concentration of CRP (log*C*_CRP_*,* ng/mL) in the range of 10 pg/mL to 100 ng/mL (*I* = −1.31 log*C*_CRP_ + 6.79, *R*^2^ = 0.995) (inset in Figure 6a). The limit of detection (LOD) of 8 pg/mL is obtained at a signal-to-noise ratio of 3. The comparison between the performance of different electrochemical sensors for the determination of CRP was shown in Table 1. The LOD is lower than that obtained from DPV detection by thiol-terminated poly(2-methacryloyloxyethyl phosphorylcholine)/gold nanoparticle modified screen-printed carbon electrode (PMPC-SH/AuNPs-SPCE) [56], ECL detection based on titania nanotubes and platinum nanowire modified indium tin oxide (TiNTs/PtNWs) [57], or DPV detection based on gold nanoparticle [12]. The starting concentration in the linear range of detection was lower than that obtained with DPV detection using branched polyethylenimine functionalized with ferrocene residues (PEI-Fc) [58], but higher than that obtained with EIS detection based on 11-cyanoundecyltrimethoxysilane and PAMAM dendrimers (11-CUTMS/PAMAM) [59] or square wave voltammetry (SWV) detection [60] based on 1-ethyl-3-(3-dimethylamino-propyl) carbodiimide hydro-chloride/N-hydroxy succinimide-activated 3-mercaptoproponic acid modified Au wire substrate (MPA/Au). The LOD is low enough for the determination of CRP levels associated with cardiovascular diseases (<1, 1–3, and >3 μg/mL for low, moderate, and high cardiovascular risks) [9] and acute myocardial infarction (>10 μg/mL) [12] in serum samples.

### 3.5. Selectivity, Reproducibility, and Stability of the Developed Immunosensor

Biological samples such as serum often contain a large amount of protein. To examine the selectivity of the constructed immunosensor, the electrodes were incubated with different proteins including procalcitonin (PCT), serum amyloid A (SAA), alpha-fetoprotein (AFP), carcinoembryonic antigen (CEA), and CRP. The electrochemical signal of the obtained electrode was measured after the immunosensor was incubated with each protein or its mixture. As shown in Figure 6b, the electrochemical signal of the probe on the electrode significantly changed only when it reacted with CRP or the protein mixture containing CRP, indicating good selectivity of the developed immunosensor. The reproducibility and storage stability of the constructed immunosensor electrodes were investigated. Five electrodes were prepared in parallel in the same batch. The relative standard deviation (RSD) for the detection of CRP (10 ng/mL) was 2.9%, indicating good reproducibility. When the immunosensor was stored in a refrigerator at 4°C, 91% of the original performance for CRP (10 ng/mL) after one week of storage was obtained, suggesting good stability of the immunosensor.

### 3.6. Detection of CRP in Real Sample

To evaluate the application potential of the developed immunosensor, human serum was selected as a real sample and the concentration of CRP in human serum was determined by the standard addition method. As shown in Table 2, the recoveries of the immunosensors ranged from 97.6% to 102% with low relative standard deviations (less than 4.1%), proving good reliability and accuracy.

## 4. Conclusions

In this work, an immunosensor for sensitive electrochemical detection of C-reactive protein (CRP) was fabricated based on the modification of a screen-printed carbon electrode (SPCE) by nanochannel array using in situ generated electrochemically reduced graphene (ErGO) as a conductive adhesive layer. With amino-containing siloxanes as precursors to rapidly grow vertically-ordered mesoporous silica nanomembrane film with amino groups (NH_2_-VMSF). During the growth of NH_2_-VMSF, graphene oxide on the electrode surface is in situ reduced to ErGO, which increases the stability of NH_2_-VMSF while providing a conductive surface. The covalent immobilization of the immune-recognitive antibody was achieved after the outer surface of NH_2_-VMSF was converted into an aldehyde-based surface using a bifunctional group of glutaraldehyde. After the antigen-antibody forms an immune complex, it will hinder the diffusion of the probe in the solution and generate a gated electrochemical signal. Based on this mechanism, sensitive electrochemical detection of CRP can be achieved. The constructed immunosensor possesses good selectivity, reproducibility, and stability. The detection of CRP in human serum has high reliability. Since SPCE is a low-cost and disposable electrode, the developed immunosensor provides an effective strategy for a convenient and sensitive determination of CRP.

## Figures and Tables

**Table 1:** Comparison between the performance of different electrochemical sensors for the determination of CRP.

Electrode Material	Detection Method	Construction Method	Linear Range (ng/mL)	LOD (ng/mL)	Ref.
PMPC-SH/AuNPs-SPCE	DPV	Label-free	5–5000	1.6	[56]
TiNTs/PtNWs/ITO	ECL	Label-free	0.05–6.25	0.011	[57]
AuNPs/SPGE	DPV	Label-based	10–15,000	1.5	[12]
GCE/PEI-Fc/Ab/CRP	DPV	Label-free	1–5×10^4^	0.5	[58]
11-CUTMS/PAMAM	EIS	Label-free	2.1 × 10^−5^–6.148 × 10^−3^	3.4 × 10^−7^	[59]
MPA/Au substrate	SWV	Label-free	5 × 10^−6^–2.2 × 10^−4^	2.25 × 10^−6^	[60]
Ab/GA/NH_2_-VMSF/ErGO/SPCE	DPV	Label-free	0.01–100	8 × 10^−3^	This work

**Table 2:** Determination of CRP in human serum samples.

Sample	Spiked (ng/mL)	Found (ng/mL)	Recovery (%)	RSD (%)
Human serum ^a^	0.100	0.102	102	4.1
	1.00	0.976	97.6	3.3
	100	99.1	99.1	3.8

^a^ Samples were diluted by a factor of 50. The indicated concentration is the concentration of CRP after dilution. The error bars represent the relative standard deviation (RSD) of three measurements.

## Data Availability

The data presented in this study are available on request from the corresponding author.
